# Is It Necessary to Integrate Evo-Devo to the Analysis and Construction of Artificial Emotional Systems?

**DOI:** 10.3389/fnbot.2022.728829

**Published:** 2022-05-31

**Authors:** Jorge Luis Hernández-Ochoa, Francisco Vergara-Silva

**Affiliations:** ^1^Posgrado en Filosofía de la Ciencia, Universidad Nacional Autónoma de México, Mexico City, Mexico; ^2^Laboratorio de Teoría Evolutiva e Historia de la Ciencia (Jardín Botánico), Instituto de Biología, Universidad Nacional Autónoma de México, Mexico City, Mexico

**Keywords:** artificial intelligence (AI), cognitive science, artificial emotional systems (AESs), embodiment, evolutionary developmental biology (evo-devo), Extended Evolutionary Synthesis (EES)

## From Turing to Developmental Robotics and Artificial Emotional Systems

Artificial intelligence (AI) is a cluster of research endeavors subtended by multiple interrelated theoretical frameworks and material practices, which in turn has been crucial for the interdisciplinary conceptualization and modeling of cognitive processes (Boden, 2014; Varela et al., 2016). Alan Turing's seminal paper from 1950 contributed with important foundations for AI as an academic field. In that work, Turing famously posed the question “can machines think?”, as well as a brilliant intuition concerning the role that ontogeny could play in scientific —and even philosophical— considerations about the nature of the mind. Briefly stated, for Turing (1950) the simulation of a child's set of mental processes that undergoes ontogenetic development should be modeled instead of its adult, synchronic or “non-ontogenetic” version. This change of perspective might turn out to be instrumental for the understanding of the brain-mind relationship, and therefore “cognition”, according to received views in standard contemporary cognitive science (Boden, 2006).

The relatively recent proposal of “developmental robotics” (DevRob) seems to derive directly from the pioneering vision of Turing. In her foreword to the seminal volume on the subject by Cangelosi and Schlesinger (2015), Linda Smith states that developmental robotics “is based on the premise that principles of developmental process are the key to engineering adaptive and fluid intelligence”, although “the promise of this idea is not yet fully realized”. Smith (2015, pp. ix–xii) identifies a number of “fundamental aspects” of human ontogeny—all of them well contained in the traditional domain of developmental psychology—which presumably would be “better understood through developmental robotics”. Following Smith's classification, these elements of human ontogeny can be briefly summarized in these terms:

(i) Extended immaturity: human beings are precocial organisms (Rosenberg, 2021). After birth, we are highly dependent on the care of members of the group into which we are born. The success of each stage of our development and learning, will depend on the scaffolding (e.g., educational institutions) provided by the culture to which we belong.
(ii) Activity: learning and acquiring different kinds of knowledge occur when organisms actively explore the world (Piaget, 1953; Lungarella et al., 2003). Understanding the ontogeny of sensorimotor schemes and their link with the environment is necessary to account for the emergence of cognition.
(iii) Overlapping tasks: perception, action and perceptual experience are connected (Hutto and Myin, 2012). In other words, organisms have multimodal and global experiences, through different ontogenetic processes and mechanisms.
(iv) Ordered tasks: this aspect points out to systematic stages of developmental sensorimotor experience that animals undergo along their ontogeny. Organisms acquire sensorimotor schemes that will allow them to have the typical behavior of their species; Held and Hein (1963), for instance, showed what happens when a group of cats are deprived of sensorimotor stimulation.
(v) Degeneracy: “Structurally variable but functionally equivalent networks are an example of degeneracy” (Sporns, 2010; p. 68). This characteristic is pertinent because different neural networks can overlap partially to support some cognitive function.
(vi) Cascades: the concept “developmental cascade” refers to different sensory stimuli that an organism experiences along his life (Byrge et al., 2014). This claim is important for early development because, across this cascade of information, unique differences between mature organisms are generated.
(vii) Individualism: Organismal identities play an important role in contemporary scientific explanation; they are irreducible to genes, population dynamics or brain areas. This point is relevant in both contemporary biology and cognitive sciences (see, e.g., Thompson, 2010; Nicholson, 2014).

Interestingly, and perhaps unsurprisingly, some of these concepts have a more-than-casual resemblance to a number of items in the long list of research concerns of evolutionary developmental biology (or “evo-devo”, for short). Evo-devo is an increasingly mature subfield of contemporary evolutionary biology which focuses on the bi-and multidirectional influences that evolution (understood primarily as the phylogenetic diversification of body plans and other predominantly structural-functional features characteristic of higher taxa) can have on ontogenetic development, and viceversa (Müller, 2007a; Arthur, 2011). As a conceptual elaboration of evo-devo, a supplementary approach that explicitly incorporates an ecological dimension to the evolution-ontogeny interactive dyad is known as “eco-evo-devo” (Gilbert and Epel, 2015; Sultan, 2015). However, Cangelosi and Schlesinger (2015) only briefly go through the basics of what they call “evolution-development, phylogenesis/ontogenesis integration”, leaving much room for elaboration on what else could DevRob gain from incorporating the insights of evo-devo and eco-evo-devo into its conceptual framework.

In line with the above, we consider that the inclusion of (eco-) evo-devo could benefit the study of artificial emotional systems (AESs), which represent a still neglected dimension in the area of “embodied cognitive robotics” (Lara et al., 2018; see also Hernández Ochoa, 2017). Some experts in this field have tried to model and implement AESs in their agents, with three main objectives, all related to emotions: (i) recognition, (ii) expression, and/or (iii) production of emotions. Researchers in the human-robot interaction (HRI) have studied the first two tasks, with their agents being used, for example, in rehabilitation therapies for children with autism (Dautenhahn and Werry, 2004) or in pedagogical applications (Karahoca et al., 2011). Regarding the third task, which might be slightly difficult to differentiate from the second, specialists have considered a central objective to study not only external characteristics but also internal ones (Arbib and Fellous, 2004; Fellous, 2004; Parisi, 2004, 2011; Parisi and Petrosino, 2010). According to these authors, for an artificial agent to have emotions *sensu stricto* it is necessary that a mechanism linked to its bodily states guides its actions, its decisions, and allows it to achieve better performance within its environment. In this context, it is claimed that if these artificial agents interact in the world through internal mechanisms—for example, homeostasis—that regulate their behavior, a better understanding of the role of the underlying mechanisms of emotions in the study of cognition will be achieved (see, for instance, Coutinho et al., 2005; Parisi and Petrosino, 2010; Schneider and Adamy, 2014). Nevertheless, the absence of an evo-devo approach in these two subjects is noteworthy.

## History and Philosophy of (Eco-)Evo-Devo, the “Extended Evolutionary Synthesis” (EES), and “Embodied AI”: The General Context

Since the 1990s, after the “homeobox gene cloning boom” of the previous decade, evo-devo has been predominantly linked to empirical (as well as formal modeling-based) investigations of gene regulatory networks (GRNs), usually analyzed in animal or plant model systems for tackling homology-related problems and other big, long-standing questions in comparative biology (see Amundson, 2005; Arthur, 2011; Wagner, 2014). These research interests are directly linked to the onset of a “golden age” of interactions between molecular genetics and developmental biology, during which the emphasis fell upon the analysis of expression patterns of “master regulatory genes” (as Walter Gehring used to call them) of cell differentiation and other developmental processes in *Drosophila* and a few other experimentally tractable insect species. These studies, replicated in a growing list of invertebrate and vertebrate animal models, supported the proposal of the “(developmental) genetic toolkit” concept and emerging general hypotheses on the genetic basis of morphological evolution largely dependent on changes in cis-regulatory elements of GRNs (e.g., Carroll, 2008). In parallel, a number of previous, long-term, integrative research lines in biology which could be considered the “core evo-devo” of that particular historical period did not refer to GRNs, or rested upon the molecular genetics of developmental processes. Instead, these investigations focused on issues such as the dynamics of interactions between tissues (e.g., Gerd Müller's work on heterochrony and fusions in vertebrate limbs), the developmental interactions that explain the conservation of morphological stages (e.g., Klaus Sander's “phylotypic stage” models), and other topics related to ontogenetic modularity and evolutionary innovation (as approached by Gerhard Schlosser, Shigeru Kuratani, and many other researchers of similar empirical and theoretical inclinations). These elaborations were in turn connected to the “developmental constraints vs. selection debate” that was central during the famous 1981 Dahlem conference in which most of the founders of “organismic evo-devo” were present (for an excellent historical review of this episode, see Love, 2015a; for further, overall context on the historiography of evo-devo, see Raff, 1996; Amundson, 2005; and Laubichler and Maienschein, 2007). Additionally, the related notions of “self-organization” and of generic physical mechanisms influencing the ontogeny of organismal form with independence from genetic controls—as attested in many publications over several decades by Stuart Kauffman and Stuart Newman, respectively—constitute yet another salient theoretical platform in the organism-centered tradition that ultimately supports the thoroughly integrative evo-devo enterprise (Love, 2015b; Nuño de la Rosa and Müller, 2021).

On the basis of this highly diversified disciplinary horizon, evo-devo has continued to establish alliances that eventually led to its participation as a major player in the most recent round of theoretical debates in international evolutionary biology, mainly as part of the so-called “Extended Evolutionary Synthesis” (EES) initiatives which coalesced in the last 15–20 years (Pigliucci and Muller, 2010; Laland et al., 2015; Fábregas-Tejeda and Vergara-Silva, 2018). Along with niche construction theory (NCT; Odling-Smee et al., 2003), evo-devo partially prefigured and therefore contributed greatly to the definition of the two main conceptual tenets of the EES: (a) the centrality of the organism, and (b) reciprocal causation (Laland et al., 2015). Given its close connection with decades-old ecological researches on phenotypic plasticity, the already mentioned theoretical configuration of “eco-evo-devo”, which relates ontogenetic development and environmental conditions —to produce new phenotypes or disease susceptibilities, for instance— leads the way to complement older, “Modern Synthesis”-associated areas of interest in evolutionary studies, such as “adaptive dynamics,” as it could clarify (with the occasional support of mathematical models) how environmental feedback loops occur in the interaction between populations and their surrounding living conditions (Metz et al., 2008; Kisdi and Geritz, 2016; Lion, 2018). Eco-evo-devo has itself recently been expanded by researchers pursuing a biological definition of “agency” (Sultan et al., 2022), but (intriguingly) not yet with a clear overlap with cognitive science, AI, or robotics concerns. For readers not familiar with the basic notions currently associated to recent discussions in evolutionary biology, coming from evo-devo, eco-evo-devo, and the EES, we provide a summary in Table 1.

**Table 1:** Recent progress in evolutionary biology originated from evo-devo, eco-evo-devo, and the “Extended Evolutionary Synthesis” (EES).

**Aspect/concept**	**Description**
Central evolutionary mechanisms/processes	Natural selection and niche construction as complementary (symmetrical) influences linking organisms and environments; “organisms” are decomposable into genetic, developmental, and behavioral elements
Influence of ontogeny in evolution	Ontogenetic constraints reconfigured as “developmental bias”, fundamental for evolvability (i.e., as facilitators of certain evolutionary trajectories)
Heredity/inheritance	Inclusive: genetic (“classic”), plus epigenetic, ecological, and behavioral/cultural/symbolic
Status of organisms as “agents”	Organisms are active subjects that modify, transform, and inherit their environments; “agency” as capacity to regulate their own persistence, maintenance, and function
Phenotypic plasticity	Inherent property of the developmental process; as important as natural selection, influencing novelty and evolvability

*Based on Laland et al. (2015); see also Müller (2021) and Sultan et al. (2022)*.

How are all of these conceptual and empirical advances in current evolutionary biology relevant to “embodied perspectives” in the field of robotics, in connection to cognitive science research projects specifically connected to AESs? More specifically: are robotics- and AI-oriented specialists already aware of the interesting opportunities that could be opened for their research projects after the adoption of this renewed eco-devo-evolutionary frameworks? Authors in the burgeoning area of “embodied AI” have recently insisted on the importance of taking seriously which role should actual physical, bodily implementations play in the construction of artificial systems—iconically, robots, but also other agents that display diverse degrees of autonomy—whose integrated properties (could) give rise to legitimate cognitive phenomena. We argue that any consideration of the ways in which such instantiations might achieve adaptive states through their participation in the construction of niches would benefit from the introduction of NCT-related or (eco-)evo-devo-derived concepts. At the same time, theoretical trends which place “embodiment” as a core concern signal a shift away from the canonical, classical “computationalist/functionalist/representationalist” vein that used to define traditional cognitive science and AI. These trends explicitly refer to the need to clarify —for the cognitive science/AI/robotics audience— “what is an organism?”, and are therefore compatible with EES notions and other recently elaborated evolutionary viewpoints. According to cognitive science specialist Ziemke (2016; p. 7), embodiment-oriented cognitive science is “largely compatible with the constructivist/enactivist/interactivist view (…) of knowledge construction in sensorimotor interaction with the environment, with the goal of achieving some “fit” or “equilibrium” between internal, conceptual/behavior-generating mechanisms and the external environment”. Ziemke's view explicitly represents an interesting convergence with the renewed, deeply relational discourse of the EES. It is no wonder, then, that especially from the standpoint of “conceptual foundations of (eco-)evo-devo” research, the time seems ripe for a more intensive dialogue between contemporary, organism-centered evolutionism and situated, enactivism-laden investigations of artificially (and biologically) embodied cognitive phenomena.

This perspective is also significant from the standpoint of the history/historiography of cognitive science and behavioral psychology, as earlier researchers such as Gilbert Gottlieb had already entertained that “neural and behavioral development at any given point in time can only be comprehended fully in light of the immediate and remote developmental history of the organism” some decades ago (Gottlieb, 1973; p. 4). Additionally, enactivist-friendly, eco-evolutionary-based viewpoints are also being applied now toward the understanding of plant cognition (see Calvo and Trewavas, 2020; and references therein). Due to long-held disciplinary divisions which put the empirical life sciences in relatively distant institutional settings, few (eco-) evo-devo biologists have applied their conceptual framework in cognitive sciences; Ploeger and Galis (2011; see also Ploeger and Galis, 2021) are an outstanding exception in this regard. These authors have emphasized the importance of this interdisciplinary integration to clarify the workings of the mind. In their papers, they have exposed some points of intersection between evo-devo and the cognitive sciences —articulating, for example, what “evo-devo comparative cognitive science” or “evo-devo cognitive neuroscience” should pursue. However, the link between AESs and (eco-)evo-devo was not analyzed in depth in those articles.

## History and Philosophy of (Eco-)Evo-Devo, the “Extended Evolutionary Synthesis” (EES), and “Embodied AI”: Teaming With “Radical Embodied Cognitive Science” for the Study of AESs

**Figure 1:**
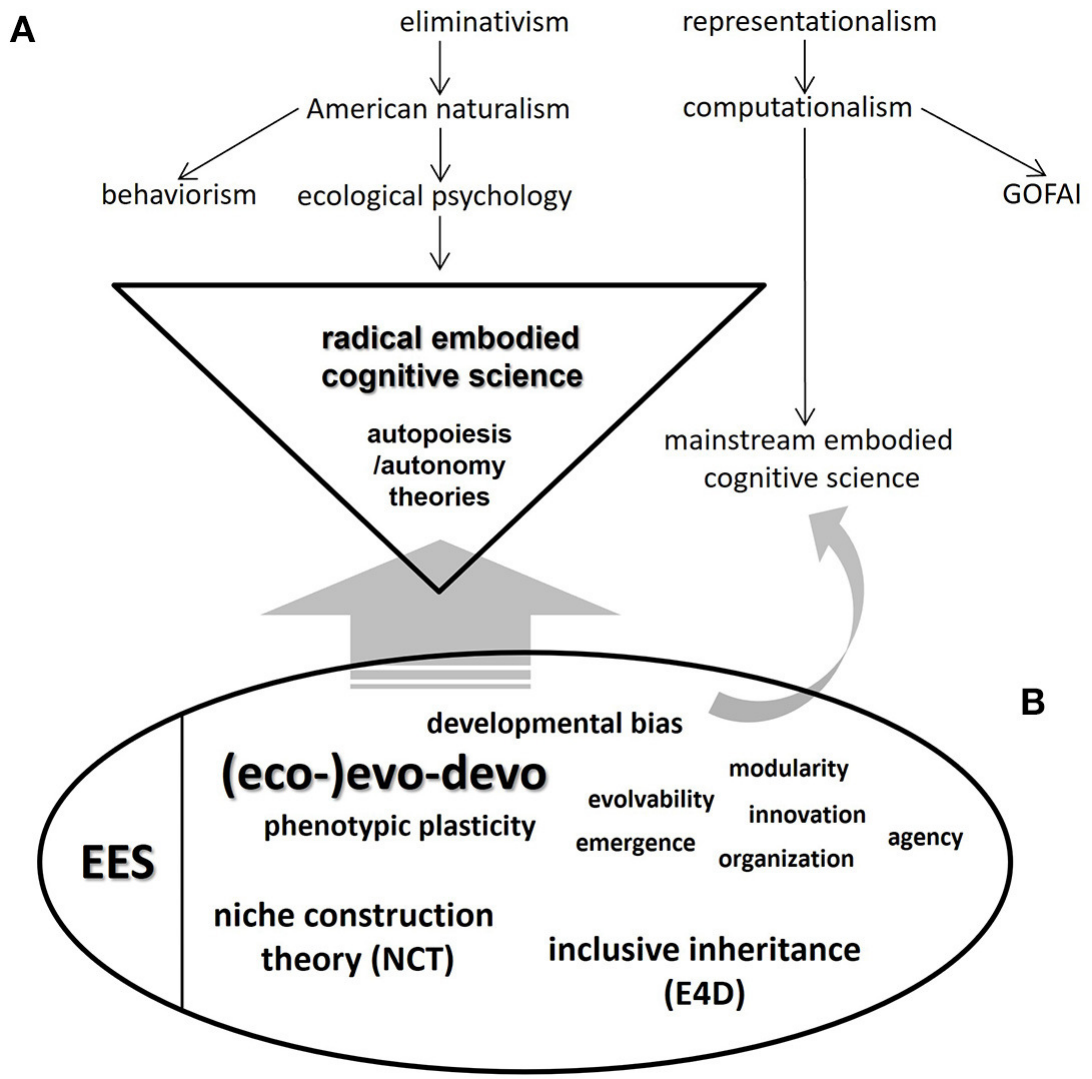
Scheme of the general relationship between **(A)** the two frameworks derived from the currently existing divide in embodied cognitive science: “mainstream” and “radical embodied cognitive science” (*sensu* Ziemke, 2016: p. 6); and **(B)** the central concepts of evolutionary developmental biology (“Evo-Devo” *sensu* Müller, 2007b; see also Müller, 2021) and the “main branches” of the Extended Evolutionary Synthesis (EES, after Laland et al., 2015). In the latter conceptual aggregation, we include (i) niche construction theory (NCT); (ii) (eco-)evo-devo, where we fuse (the study of) phenotypic plasticity-related phenomena as well as developmental bias, and a number of concepts and theoretical themes and implications of evo-devo *sensu stricto*; and (iii) inclusive inheritance. Müller (2007b) additionally defines Evo-Devo in terms of four complementary “research programs”, all of which structure our understanding of the field. “Radical embodied cognitive science” is highlighted (triangle) to stress the stronger compatibility with and/or susceptibility of influence from (broad, straight gray arrow) the (eco-)evo-devo/EES framework. A slender, curved gray arrow pointing to “mainstream embodied cognitive science” indicates a remaining, restricted potential for certain (eco-)evo-devo concepts to help in certain tasks in this relatively conservative branch of cognitive science. To emphasize the usefulness that this renewed evolutionary framework could have for the analysis and construction of artificial emotional systems (AESs) in the context of “embodied AI” research, some basic definitions are provided (see also Table 1): (a) *Developmental bias* is the “source of bias in phenotypic variation (...) which does not only constrain but also facilitate and direct evolution” (Laland et al., 2015, p. 5); (b) *developmental* (or *phenotypic*) *plasticity* is “the capacity of an organism to change its phenotype in response to the environment” (Müller, 2007b; Laland et al., 2015, treats this concept as the basis for his conception of “eco-evo-devo”); (c) *modularity* is a feature of organismal parts/structures “pervasive at all levels (...), from the genetic to the developmental, anatomical and behavioral” that distinguishes them in terms of “greater internal than external integration” as well as “repetitiveness (...) persistence and reuse” (Müller, 2007b, p. 944); (d) *evolvability* is “ the intrinsic potential of a given lineage to produce heritable phenotypic variation”, related to “variational capacities of genomes (as) functions of the developmental systems in which they are embedded” (Müller, 2007b, p. 946); (e) *emergence* “refers to phenomena outside the scope of variation, in particular to the modes of origination, innovation and novelty in phenotypic evolution” (Müller, 2007b, p. 946); (f) *innovation* refers to “instances of novelty”, due to “the redeployment of existing regulatory circuits” or “the mechanisms of epigenetic causation” (Müller, 2007b, p. 945); (g) *(phenotypic) organization* emphasizes that “the causal basis for phenotypic form resides not in population dynamics or (...) molecular evolution, but instead in the inherent properties of evolving developmental systems”, signaling an explicit non-selectionist, developmentalist explanatory style that feeds back upon corresponding conceptions of classical comparative biology such as homology, homoplasy, and body plans (Müller, 2007b, pp. 947–948); and (h) *(biological) agency* is “the capacity of a system to participate in its own persistence, maintenance, and function by regulating its own structures and activities in response to the conditions it encounters” (Sultan et al., 2022, p. 4; this is an outstanding post-EES development which suggests additional links that this evolutionary framework might have with cognitive science and “embodied AI” research). In addition, *inclusive inheritance* is roughly equivalent to the system of “supragenetic heredity channels” postulated by Jablonka and Lamb (2014) in their “evolution in four dimensions” (E4D) model. As stated in the main text, “centrality of the organism” and “reciprocal causation” are the two core principles underlying the (eco-)evo-devo/EES conceptual system depicted in the figure. Upper scheme ([A]; i.e., genealogy of current notions of embodied cognitive science) adapted from Ziemke (2016), after Chemero (2009).

So far, we have hinted at how DevRob, AESs and “enactive/embodied cognitive science” might obtain tangible benefits from further disciplinary interactions with current evolutionary theorizations that locate ontogeny and the organism-environment duality as crucial concerns. A full, in-depth elaboration of such interactions —again, both conceptually and in history of science terms— would therefore seem to be in order. In Figure 1, we present an initial, firm step in that direction, taking advantage of the useful scheme of Ziemke (drawn after Chemero, 2009) in his already cited work (2016). As reproduced in our own version of that scheme (upper right part of Figure 1), Chemero (2009, p. 30) and Ziemke (2016, p. 6) depict a genealogy of major trends in cognitive science leading to the two main branches of embodied cognitive sciences: “radical” (left side) and “mainstream” (right side). In the bottom left side of our graphic proposal, we present a highly simplified picture of the main theoretical themes and implications of “Evo-Devo” (in the nomenclature of Müller, 2007b; largely equivalent to the notion of (eco-)evo-devo used here), as they in turn relate to the conceptual branches of the “Extended Evolutionary Synthesis” (EES *sensu* Laland et al., 2015). “Developmental bias” —i.e., the central conceptual contribution of evo-devo *sensu stricto* to the EES (Laland et al., 2015, p. 3)— occupies a visible place in the remaining cloud of (eco-)evo-devo concepts.

From the standpoint of the history and philosophy of evolutionary biology, a final observation should be made in relation to the “core notions” of (eco-)evo-devo, as summarized above: in our figure, we have not included key concepts related to GRNs. This is because the version of (eco-)evo-devo that we consider most compatible with embodied cognitive sciences is not the “gene-centric” one, championed by researchers working mainly in the “evolutionary developmental genetics programme” *sensu* Müller (2007b, p. 943). Interestingly, an analogy could be drawn between this arena of mostly empirical, laboratory and model systems-based research and the dominant status of mainstream embodied cognitive science. In this regard, a stronger compatibility between the (eco-)evo-devo/EES framework and radical embodied cognitive science is claimed (broad, straight gray arrow in Figure 1), still leaving some room for restricted influences of certain (eco-)evo-devo concepts (e.g., emergence; modularity; organization; among others) upon the corresponding mainstream side (slender, curved gray arrow in Figure 1).

The ideas of Ziemke (2016) are of additional interest for their emphasis on the “hard problem” status that characterizes the analysis and construction of AESs. Here we refer to implementations (of an abstract nature in standard, non-embodied cognitive science/AI research; Coutinho et al., 2005; Parisi and Petrosino, 2010; Schneider and Adamy, 2014) in which “states (lead to) increase the correctness and effectiveness of the motivational decisions (of the system) by influencing the current intensity of the different motivations” (modified from Parisi and Petrosino, 2010, p. 3). We consider that, as a matter of principle and in ways that remain to be empirically studied in detail under increasingly stringent methodological criteria, most elements of the (eco-)evo-devo/EES conceptual framework (lower ellipse, Figure 1) are applicable to the study of AESs. Even under a general agreement that “robotic phenotypic plasticity” would be practically impossible unless reliable ways to build structurally (and functionally) cell-like-based materials were developed, it would be perfectly valid to physically confirm (or virtually model) the changes brought forward by embodied agents in concrete environments under the assumptions of NCT. In this respect, bringing back Conrad Waddington's work (1942, 1956) —a central precursor of that theory and many other pillars of (eco-) evo-devo and the EES (Fabris, 2021)—could be fruitful to enrich the theoretical basis for the analysis and construction of AESs. To further support the relevance of his ideas in this context, we very briefly mention here three central Waddingtonian concepts: (i) *canalization* —understood as the “adjustment of developmental reactions so as to bring about one definite end result regardless of minor variations in conditions during the course of the reaction” (Waddington, 1942; p. 563; see also Schmalhausen, 1949)—; (ii) *genetic assimilation* —i.e., a process by which “a character which had originally been an ‘acquired' one might then be said to have become genetically assimilated” (Waddington, 1956; p. 1)—; and finally (iii) *epigenetic landscape —*which this author famously presented as a graphic representation of the contingent history of the ontogenetic trajectories of biological systems (Waddington, 2014/1957). Thinking in Waddington's evolutionary key for the design of AESs could be further complemented through assessments of the character and dynamics of (modified versions of) the three supragenetic (epigenetic; symbolic; cultural) heredity channels postulated by the “inclusive inheritance” theoretical prescription of the EES, roughly equivalent to the explicitly Waddingtonian “evolution in four dimensions” (E4D) framework of Jablonka and Lamb (2014).

It should not be controversial, then, to say that treating “artificial emotional systems” (AESs) under the guide of (eco-) evo-devo/EES principles implies to conceive of them in terms of evolutionary mechanisms that have not been central in attempts based on the “standard view” of evolution, commonly held in GOFAI or other older frameworks of cognitive science —including, by the way, certain views of “adaptive dynamics of behavior” (e.g., Staddon, 2001). In this regard, Ploeger and Galis (2021) helpfully indicate further interdisciplinary connections between the wide domain of cognition studies and diverse evolutionary-oriented perspectives in the life sciences. Consequently, the adoption of contemporary evolutionary biology standpoints in current cognitive science, especially given the convergent impulse seen in fields such as “developmental robotics” (*sensu* Cangelosi and Schlesinger, 2015) and especially “radical embodied cognitive science” (*sensu* Ziemke, 2016), could pave the way for truly innovative strategies to solve recalcitrant research problems in general cognitive science and AI research —for instance, the question of “what is embodiment” (Ziemke, 2016, p. 5), and therefore in robotics. Now, could the analysis of existing AESs according to (eco-)evo-devo/EES premises, as well as the construction of new such systems under those assumptions, be a fruitful way to follow Turing's intuitions of the importance of ontogeny in the genesis of the human mind? Clearly, answering this question would demand the serious proposal of “thought experiments” and/or narratives that get us closer to an ability to engineer an actual ontogenetic model, as opposed to one that is “created” as an adult (even if it is an adult that can learn). Drawing partly from diverse sources (even science fiction films, e.g., Garland (2014) *Ex Machina*, where Ava passes the Turing test by deceiving Caleb), we finally suggest that if researchers were able to build a (physical or virtual) AES that responded to the set of ecological/ontogenetic and evolutionary mechanisms and processes exposed here, they could obtain an agent that would allow a much deeper understanding of the intricate entanglements that constitute cognition, perception and, ultimately, emotion.

## Author Contributions

All authors listed have made a substantial, direct, and intellectual contribution to the work and approved it for publication.

## Funding

JH-O is currently supported by a Consejo Nacional de Ciencia y Tecnología (CONACYT, Mexico) doctoral scholarship (661142).

## Conflict of Interest

The authors declare that the research was conducted in the absence of any commercial or financial relationships that could be construed as a potential conflict of interest.

## Publisher's Note

All claims expressed in this article are solely those of the authors and do not necessarily represent those of their affiliated organizations, or those of the publisher, the editors and the reviewers. Any product that may be evaluated in this article, or claim that may be made by its manufacturer, is not guaranteed or endorsed by the publisher.
